# Prior infections and polymorphism in NECTIN2 gene are synergistically associated with reduced hippocampal volume, a biomarker of neurodegeneration, in older women

**DOI:** 10.1002/alz.093512

**Published:** 2025-01-09

**Authors:** Svetlana Ukraintseva, Vladimir Popov, Hongzhe Duan, Arseniy Yashkin, Igor Akushevich, Konstantin Arbeev, Anatoliy Yashin

**Affiliations:** ^1^ Duke University, Durham, NC, USA, Social Science Research Institute, Duke University, Durham, NC USA

## Abstract

**Background:**

A growing body of research suggests a connection between Alzheimer’s disease (AD) and prior infections, although the mechanisms are not well understood. We recently reported a significant association between a history of adult infections and smaller brain hippocampal volume (HV), a major biomarker of neurodegeneration, in female participants of the UK Biobank (UKB). Here we further investigate this connection, taking into account the rs6859 polymorphism in the NECTIN2 gene, an established genetic risk factor for AD that is also involved in vulnerability to infections.

**Methods:**

We estimated the associations between adult history of infections and HV assessed later in life, at ages 65‐80 years, in the presence and absence of the AD risk variant (A) of rs6859, using the UKB subsample with respective information (N=11,765; 48% women). Infectious disease diagnoses were based on ICD‐10 codes. The left/right HV was measured by magnetic resonance imaging (MRI) in cubic millimeters (mm^3^) and normalized. Analysis of variance (ANOVA), Tukey’s test, Welch test, and regression were used to examine statistical significance.

**Results:**

Prior infections and carrying the AD risk variant rs6859 (A) were synergistically associated with a significant reduction in the left HV by ∼2.1% (p‐value=0.03) and in the right HV by ∼2.2% (p‐value=0.01) in women aged 65‐80 years. Results for men did not reach statistical significance.

**Conclusion:**

Results of our study suggest that prior infections and rs6859 in the NECTIN2 gene synergistically contribute to neurodegeneration in older women. A potential explanation of this effect could be that carrying the rs6859 (A) may aggravate AD‐related brain pathology triggered by infections because this genetic risk factor for AD also increases the brain’s vulnerability to infections. This hypothesis needs confirmation in biomedical research. The observed sex difference may reflect a higher vulnerability of the female brain to infection‐related factors due to hormonal or other differences between males and females, which deserves further investigation.

